# Increased Adiposity Appraised with CUN-BAE Is Highly Predictive of Incident Hypertension. The SUN Project

**DOI:** 10.3390/nu13103309

**Published:** 2021-09-22

**Authors:** Ligia J. Dominguez, Carmen Sayón-Orea, Alfredo Gea, Estefania Toledo, Mario Barbagallo, Miguel A. Martínez-González

**Affiliations:** 1 Geriatric Unit, Department of Internal Medicine and Geriatrics, University of Palermo, 90127 Palermo, Italy; mario.barbagallo@unipa.it; 2 Department of Preventive Medicine and Public Health, University of Navarra-IDISNA, 31008 Pamplona, Spain; msayon@unav.es (C.S.-O.); ageas@unav.es (A.G.); etoledo@unav.es (E.T.); mamartinez@unav.es (M.A.M.-G.); 3 CIBER Fisiopatologia de la Obesidad y Nutrición (CIBERobn), Instituto de Salud Carlos III, 28029 Madrid, Spain; 4 Public Health Institute, 31003 Navarra, Spain; 5 Department of Nutrition, Harvard TH Chan School of Public Health, Boston, MA 02115, USA

**Keywords:** hypertension, blood pressure, body fat, adiposity, CUN-BAE, obesity, overweight, diet, cohort studies

## Abstract

Overweight and obesity are growing worldwide and strongly associated with hypertension. The Clínica Universidad de Navarra-Body Adiposity Estimator (CUN-BAE) index is proposed as an optimal indicator of body fatness. We aimed to investigate the association of body fat as captured by the CUN-BAE index with incident hypertension in a Mediterranean population. We assessed 15,950 participants of the SUN (Seguimiento Universidad de Navarra) prospective cohort (63.7% women) initially free of hypertension. Participants completed follow-up questionnaires biennially. A validated 136-item food-frequency questionnaire was administered at baseline. We used Cox models adjusted for multiple confounders. Among 12.3 years of median follow-up (interquartile range: 8.3, 15.0 years), 2160 participants reported having received a diagnosis of hypertension. We observed a strong direct association between progressively higher the CUN-BAE index at baseline and incident hypertension during follow-up in multivariable-adjusted models for men and women, even after further adjustment for BMI ≥ 30 kg/m^2^, showing a significant association also in non-obese participants. For each 2-unit increase in the CUN-BAE index, hypertension risk increased by 27% and 29% in men and women, respectively. The results remained significant when considering longitudinal repeated measures of changes in body fat assessed with the CUN-BAE index among the different biennial follow-up questionnaires. Our results emphasize the importance of reducing and maintaining a low body fat to prevent hypertension.

## 1. Introduction

Data from 195 countries worldwide of the Global Burden of Disease study assessing the trends in the prevalence of overweight and obesity among adults and children between 1980 and 2015 showed that 107.7 million children and 603.7 million adults were obese in 2015 with a twofold increased prevalence since 1980 in over 70 countries and a continuous rise in most other countries [1]. High body mass index (BMI) accounted for 4.0 million deaths globally (two-thirds from cardiovascular disease (CVD)), of which near 40% occurred in persons who were not obese. Obesity prevalence, which continues to rise, represents thus a major health and economic burden [2].

There is compelling evidence to support the concept that obesity, defined as the accumulation of fatty tissue, is a causative factor in the development of hypertension [3,4,5,6,7]. The relationship of obesity and hypertension has been well defined in adults and children across both men and women [3,8]. For example, 78% of incident hypertension in men and 65% in women were attributable to excess body fat in the Framingham Offspring Study [9]. Likewise, a 5% increase in body weight was associated with 20% to 30% increase in incident hypertension [10]. BMI was a robust risk factor for incident hypertension among 82,882 women from the Nurses’ Health Study II after 14 years of follow-up. Obese women showed a five-fold higher risk of incident hypertension compared to lean women (BMI lower than 23 kg/m^2^) [11]. Accordingly, an even modest body weight reduction has been linked to decreased blood pressure values in hypertensive patients. For example, in a study in which overweight and obese adults were randomized to a weight loss intervention program vs. usual care in the Trials of Hypertension Prevention phase II study, participants who sustained a weight reduction of 4.5 kg for 30 months had a 65% reduction in their risk of developing hypertension [12].

Hypertension is a robust independent modifiable risk factor for stroke, myocardial infarction, chronic kidney disease, cognitive deterioration, and heart failure [13]. Hypertension was associated with 4.9, 2.0, and 1.5 million deaths caused by coronary heart disease, ischemic stroke, and hemorrhagic stroke, respectively [14]. The global incidence and prevalence of hypertension and, especially, its cardiovascular complications are still increasing [15]. Thus, public health preventive approaches are urgently warranted to fight the hypertension pandemic.

There are various methods for estimating the percentage of body fat (BF), including air displacement plethysmography (ADP), dual-energy X-ray absorptiometry (DXA), and magnetic resonance imaging (MRI), among others [16]. Yet, their high cost, complexity, radiation risk, and problematic transport due to their large size, among other issues, make their use greatly challenging in the usual clinical and community settings [17,18]. Therefore, diverse indices and formulas derived from anthropometric variables have been proposed in different populations to overcome these problems. Moreover, they have shown a higher correlation with BF than BMI [19,20,21,22]. In addition, these equations are simple and practical to use, accessible, and have exhibited a strong association with chronic diseases [23]. Among these equations, the Clínica Universidad de Navarra-Body Adiposity Estimator (CUN-BAE), based on BMI but also taking into consideration age and sex, was validated using ADP as a gold standard, and it has been widely used in clinical studies because of its accuracy and its association with CVD and metabolic conditions [22,23,24,25,26,27]. The percentage of fat content calculated using the CUN-BAE showed a stronger correlation with the actual amount of adipose tissue than any other anthropometric fat indicator (r = 0.89) [22]. Thus, the CUN-BAE can embody an effective instrument for identifying individuals at risk for CVDs. Only one longitudinal study has previously investigated the CUN-BAE in relation to incident hypertension [27].

We aimed to prospectively investigate the association of body fat as captured by the CUN-BAE index with the risk of incident hypertension in the Mediterranean population of the SUN (“Seguimiento Universidad de Navarra”) longitudinal project.

## 2. Methods

### 2.1. Study Design and Population

For the analyses presented here, we used data from the SUN Project, a multipurpose, prospective, longitudinal, constantly open cohort study of university graduates launched in 1999. Information on diet, lifestyles, other health risk factors, and medical disorders was collected at baseline and updated every 2 years. Further information can be found elsewhere [28,29,30].

**Figure 1:**
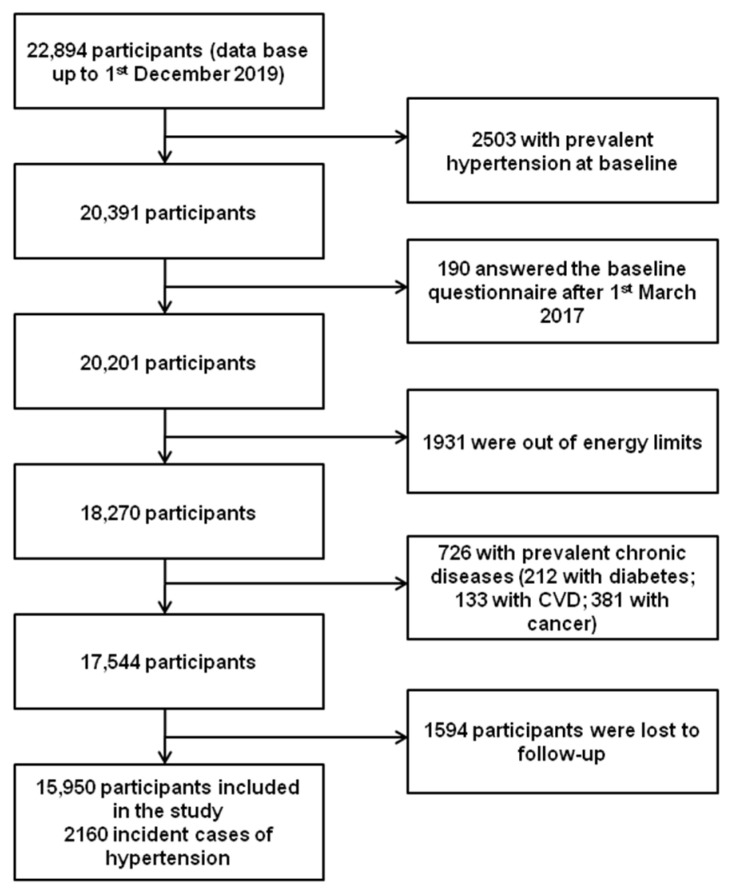
Flow-chart of participants of the SUN project to be included in the present analyses.

We performed the analyses taking in consideration the last available database as of December 1st, 2019, corresponding to 22,894 participants. We excluded from the analyses those participants with hypertension at baseline (*n* = 2503), those who answered the baseline questionnaire after 1 March 2017 (*n* = 190), those with total energy intake out of usual predefined limits [31] (*n* = 1931), those with other chronic diseases at baseline (with diabetes *n* = 212; with CVD *n* = 133; with cancer *n* = 381), and those who were lost to follow-up (*n* = 1594). The final effective sample for longitudinal analyses was of 15,950 participants with an overall retention rate of 90.91 (90.91% of participants returned ≥ 1 follow-up questionnaire). During follow-up, 2160 participants reported having received a new diagnosis of hypertension (Figure 1).

### 2.2. Ethics

All participants received written information regarding their specific data to be requested by future questionnaires, the research team’s upcoming feedback, and the protection of their privacy. They were also informed about their right to reject participating in the SUN project or to remove their participation consent at any time with no reprisal, following the indications of the Declaration of Helsinki. The compilation of the questionnaire sent at baseline in a totally voluntary manner was considered to indicate informed consent. The Research Ethics Committee of the University of Navarra confirmed this procedure of requesting participants’ informed consent (Project identification code 2001_30).

### 2.3. CUN-BAE

We calculated the CUN-BAE index with the equation proposed by Gomez-Ambrosi et al. [22]:

BF% = −44.988 + (0.503 × age) + (10.689 × sex) + (3.172 × BMI) − (0.026 × BMI^2^) + (0.181 × BMI × sex) − (0.02 × BMI × age) − (0.005 × BMI^2^ × sex) + (0.00021 × BMI^2^ × age), where age was measured in years and sex was codified as men = 0 and women = 1.

### 2.4. Incident Hypertension

The main outcome of the present analyses was a self-reported medical diagnosis of hypertension in any of the follow-up questionnaires; this appraisal has been previously and repetitively validated [32,33,34]. One of the validation studies was conducted among a subsample of participants to the SUN project who disclosed a diagnosis of hypertension formulated by a physician, was on treatment with antihypertensive medications, or both in any of the follow-up questionnaires. In this sub-sample, two independent medical doctors (unaware of what was stated by participants) performed direct home-based assessment of blood pressure in those participants who had indicated a diagnosis of hypertension or a hypertension-free status. Hypertension, as defined by the 2018 European guidelines (values higher than 140/90 mmHg) [35], was confirmed in 82.3% (95% confidence interval (CI) 72.8, 92.8%) of the self-reported hypertension cases reported in the questionnaires. Among participants who did not denote self-reported hypertension in the questionnaires, 85.4% (95% CI 72.4, 89.1%) were not found to be hypertensives [32]. For the present analyses, when there was missing information on the date of a participant’s self-reported hypertension diagnosis, we used the mid-point between the date of compilation of the follow-up questionnaire indicating a self-reported medical diagnosis of hypertension and the previous follow-up questionnaire to impute the date of diagnosis.

### 2.5. Other Covariates

In the multivariable analysis, we adjusted for several variables that could have a potential role as confounders in the relationship between adiposity and hypertension to estimate the independent contribution of the CUN-BAE score to this relationship, always stratifying the analyses for men and women. The methods used for the assessment of covariates in the SUN project have been previously reported many times in sufficient detail [28,29,30]. The variables included were: body weight changes, marital status, dietary and other lifestyle risk factors (such as total energy intake, following special diets, snacking between meals, sugar-sweetened beverage consumption, adherence to Mediterranean diet [36], minerals intake (sodium, calcium, magnesium, and potassium), smoking habit in three categories and pack-year, physical activity, hours/day spent watching television, and years of university education), as well as other confounding risk factors (such as family history of hypertension, hypercholesterolemia, and/or hypertriglyceridemia at baseline, years of entrance to the cohort, and analgesic consumption). Adjusting for the adoption of a special diet refers to the possibility that the participant has followed particular diets different from ad libitum consumption, i.e., for a particular purpose or advised by a doctor or nutritionist. This variable could also be a proxy of clinical conditions not captured by other items or of subclinical conditions. Age, sex, and BMI were already included in the CUN-BAE index calculation. It is well-known that body fat and incident hypertension increase with age. Nevertheless, because the estimation of body fat with the CUN-BAE index already includes age in its calculation, controlling the models for age would induce over adjustment because age is a component of the independent variable in the models, and the results would be misleading [37]. In fact, the Pearson correlation coefficient between age and the CUN-BAE score was = 0.32. For assessing physical activity, we used a questionnaire that has been previously validated with objective measurements by means of a triaxial accelerometer (RT3 Triaxial Research Tracker) as reference (Spearman correlation coefficient of 0.51; *p* < 0.001) [38]. Physical activity was expressed in metabolic equivalent tasks (METs-h/week) calculated as the time spent at each activity in hours/week multiplied by its usual energy expenditure [39]. Self-reported weight and height to calculate BMI have been formerly validated in a subsample of the SUN cohort [40].

### 2.6. Statistical Analyses

The description of baseline characteristics of the sample was performed computing means and SDs for continuous variables and proportions for categorical variables across even quartiles of body fat as captured by the CUN-BAE index, separately for men and women. We calculated the follow-up time from the date the participant returned the baseline questionnaire to the date in which incident hypertension was diagnosed or to the date when the participant returned the last follow-up questionnaire. We calculated the incidence rate of hypertension across baseline quartiles of the CUN-BAE index, and calculated hazard ratios (HRs) and 95% CI with Cox proportional hazards models using the lowest quartile of the CUN-BAE index as the reference. We tested the proportional-hazards assumption with the test based on Schoenfeld residuals after fitting the model. We also analyzed repeated measurements of CUNBAE-index calculated with updated information in the follow-up questionnaires in time-dependent Cox models. We first estimated HRs without any adjustment (crude); we then performed adjustments for multiple confounders, separately for men and women, as follows: (1) Model 1: HRs were adjusted for marital status, body weight changes, years of university education, smoking, pack-year, physical activity, television watching, family history of hypertension, total energy intake, adherence to the Mediterranean diet (0 to 9 points), according to reference [36], sugar-sweetened beverages, between-meal snacking, adoption of special diet, dietary sodium, dietary potassium, dietary calcium, dietary magnesium, hypercholesterolemia, hypertriglyceridemia, use of analgesic drugs, and year of entrance to the cohort; (2) Model 2: HRs were additionally adjusted for BMI higher than 30 kg/m^2^. We used the median of the CUN-BAE index quartiles as a continuous variable to calculate the significance of the linear trend tests. We then used multivariable-adjusted HRs estimates to calculate the association of the CUN-BAE index increase by two and 10 units with the incidence of hypertension. We also performed analyses considering the risk of incident hypertension for each increase of one standard deviation (z-BF) of body fat assessed with the CUN-BAE index as a continuous variable. Kaplan–Meier failure function hazards estimates were plotted for hypertension incidence according to body fat as captured by the CUN-BAE index at baseline. We then performed several sensitivity analyses estimating the fully-adjusted HRs for the comparison between the highest with the lowest quartile of body fat captured by the CUN-BAE index and its association with incident hypertension after changing several assumptions: (1) in order to examine if using a different method of estimating energy intake vs. the one we originally used [31] we repeated the analyses considering energy intake between percentages 1 and 99 to exclude extreme values of energy intake; (2) censoring the follow-up time of participants at 14 or more years; (3) excluding participants in whom the diagnosis of incident hypertension was formulated during the first 2 years of follow-up; (4) including only participants younger than 40 years; (5) including only participants younger than 60 years. We also performed analyses estimating the fully adjusted HRs for the comparison between the highest with the lowest quintile of body fat captured by the CUN-BAE index and its association with incident hypertension. We assessed the interaction between sex and CUN-BAE index quartiles using likelihood ratio tests in the fully adjusted Cox models. We introduced a product term with both sex and the CUN-BAE index quartiles as continuous variables in this term. We used Stata software package version 15 (Stata Corp) for performing the analyses. All *p*-values were 2-tailed and considered significant for *p* < 0.05. Values in the text are means ± SDs unless otherwise indicated.

## 3. Results

### 3.1. Participants

Among 184,798 person-years of follow-up (median follow-up time: 12.3 years; interquartile range: 8.3, 15.0 years) from 1999 to 2019 there were 2160 cases of incident hypertension in the SUN cohort. Table 1 shows the characteristics of participants at baseline, including demographic, anthropometric, lifestyle parameters, food consumption, and nutrient intake, according to quartiles of body fat captured with the CUN-BAE index. Both men and women in the highest quartile of the CUN-BAE index were more likely to be older, married, and smokers, had higher BMI, higher alcohol consumption, higher family history of hypertension, higher personal history of hypercholesterolemia and hypertriglyceridemia, higher use of analgesic drugs, and were more likely to follow a special diet compared to those in the lowest quartile. Men in the highest quartile of the CUN-BAE index had lower levels of leisure-time physical activity compared to those in the lowest quartile.

### 3.2. Body Fat and Incident Hypertension

As shown in Table 2, we observed a strong direct association of progressively higher body fat in quartiles of the CUN-BAE index at baseline with incident hypertension during follow-up in the crude model and after multivariable adjustments (Model 1) in both men and women with significant linear trends. Even after further adjustment for BMI ≥ 30 kg/m^2^ (Model 2) the results remained significant. The risk of incident hypertension for each 10-unit increase in the CUN-BAE index was over three-fold (HR 3.35; 95% CI 2.65, 4.24). Moreover, even a smaller change (each 2-unit increase in the CUN-BAE index) was strongly significant in the fully adjusted multivariable adjusted analyses in both men and women. Considering longitudinal repeated measures with changes in body fat assessed with the CUN-BAE index among the different follow-up questionnaires, the results were of similar magnitude for crude values, Model 1, Model 2, linear trends, 10-unit increase, and 2-unit increase in the fully adjusted models for both men and women (Table 2).

**Figure 2:**
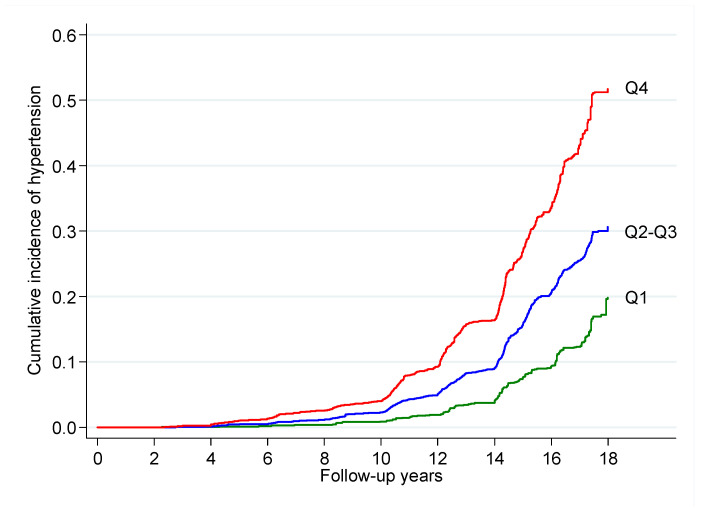
Cumulative incidence of hypertension during follow-up for the lower quartile (Q1), the second and third quartiles combined (Q2–Q3), and the fourth quartile (Q4) of body fat as captured by the CUN-BAE index after adjustment for potential confounders among men and women in the SUN (“Seguimiento Universidad de Navarra”) cohort, 1999–2019.

Figure 2 shows the cumulative incidence of hypertension during follow-up for the lower quartile (Q1), the second and third quartiles combined (Q2–Q3), and the fourth quartile (Q4) of body fat.

Considering the risk of incident hypertension for each increase of one standard deviation (z-BF) of body fat assessed with the CUN-BAE index as a continuous variable, we observed a significant increased risk for crude values and multivariable-adjusted Model 1 and Model 2, both for incident hypertension considering the CUN-BAE index at baseline, as well as considering longitudinal repeated measures of changes in body fat assessed with the CUN-BAE index in the different follow-up questionnaires for men and women (Table 3).

### 3.3. Interaction between Sex and the CUN-BAE Index

When we assessed the interaction of potential modifying effect of sex by the CUN-BAE index in the fully-adjusted models, we did find a statistically significant interaction when considering quartiles of the CUN-BAE index (*p* = 0.0017), although for z-BF the interaction was borderline significant (*p* = 0.067).

### 3.4. Sensitivity Analyses

Table 4 shows the results of several sensitivity analyses for the association of body fat, as captured by quartiles of the CUN-BAE index, and incident hypertension. This association remained strongly significant for both men and women in the different scenarios that we considered: (1) considering percentiles 1 to 99 as limits for allowed total energy intake; (2) censoring the follow-up time of participants at 14 years; (3) not including early incident hypertension (diagnosis during the first two years of follow-up); (4) including only participants aged below 40 years; (5) including only participants aged below 60 years; (6) considering quintiles of body fat as captured by the CUN-BAE index (comparing the highest quintile to the lowest).

## 4. Discussion

The present results from analyses on data of the SUN Project, a well-characterized, prospective, and large cohort of Spanish university graduates, showed that body fat as captured by the CUN-BAE index was positively and strongly associated with incident hypertension after a long-term follow-up, independently of numerous potential confounders that were considered in the fully-adjusted multivariable models. The risk of incident hypertension was also independently associated with the CUN-BAE-estimated body fat even after adjusting for obesity considering the classical cut-off point of BMI of 30 kg/m^2^, which suggests that progressively higher adiposity is independent of being obese for long-term incidence of hypertension. The results remained significant after several sensitivity analyses as well as when we considered repeated measures of the CUN-BAE index during follow-up. Only one longitudinal study has previously reported a significant association of the CUN-BAE index with incident hypertension [27]. This is the first time that the CUN-BAE index has been investigated in relation to incident hypertension in a longitudinal study in a Mediterranean population.

### 4.1. Previous Investigations

There is compelling evidence supporting that increased adiposity manifested as obesity is closely linked to hypertension [3,4,5,6] in men, women, and children [3,8]. Most hypertension cases in the Framingham Heart Study [10] and the Framingham Offspring Study [9] were imputable to excess body fat. This was confirmed in the prospective Nurses’ Health Study II, where elevated BMI was the strongest risk factor for incident hypertension [11]. BMI has been widely used as a biometric parameter for defining a normal, under, or overweight/obesity condition in relation to height. While BMI alone may correlate with body fat percentage, it has a low predictive ability [41,42,43]. Conversely, when other variables, including sex and age, are incorporated into the analyses, the accuracy significantly improves [18,21,22]. Therefore, the use of BMI in isolation is controversial because it does not consider the influence of other key variables for estimating body composition, such as age or sex [41]. The same value of BMI can be associated with a widespread body fat percentage (from healthy to pathological values), rendering difficult the clinical assessment [44]. In fact, BMI may largely increase as adiposity increases, but differences in body composition make the correlation weak. For example, a person with greater muscle mass or larger bones will have a higher BMI, which would not represent increased body fat. Hence, BMI is a useful indicator of overall fitness in epidemiological studies but a poor instrument for determining health outcomes on an individual basis. However, the methods used to quantify body fat directly are expensive and not routinely available in daily clinical practice [17,18]. For this reason, several equations have been developed, including the CUN-BAE index, which has previously shown accuracy and validity in relation to CVDs and metabolic conditions [22,23,24,25,26,27].

The estimation of body fat with the CUN-BAE index specifically in relation to hypertension has been evaluated in few studies [25,26,27,45,46]. In a cross-sectional sample of 12,122 non-institutionalized participants from the ENRICA (nutrition and cardiovascular risk in Spain) study, the CUN-BAE index was directly associated with hypertension, diabetes, and metabolic syndrome in adults independently of BMI or waist circumference, suggesting that it may help to identify persons with cardiometabolic conditions beyond BMI [25]. Another cross-sectional sample of 2354 adults with intermediate CV risk from the interMediAte RisK management (MARK) study explored the relationship of adiposity assessed with two indices, the CUN-BAE index and body roundness index (BRI), with arterial stiffness. Both adiposity measures were negatively associated with arterial stiffness, but the stiffness variability was better explained with the CUN-BAE index and BMI than with BRI [45]. A case-control study involving 47 patients with neuromyelitis optica spectrum disorder (NMOSD) and 28 patients with multiple sclerosis (MS) both at acute phase; 21 NMOSD and 25 MS patients at stable phase; and 68 age- and sex-matched healthy controls found that women with NMOSD were more prone to have increased blood pressure values and fat mass compared to MS patients. Acute myelitis was more likely to occur in NMOSD patients with high body fat at the acute phase [46]. Another cross-sectional study of 3888 participants comparing BMI and the CUN-BAE index did not find a good correlation between these two parameters. The attributable fraction for the presence of hypertension was doubled using the CUN-BAE index than using the BMI [26]. Analyses of data from 6796 participants of the prospective cohort study Hordaland Health Study from Norway found that the CUN-BAE index showed stronger associations with incident hypertension than BMI in both the total population and in sex-stratified analyses [27]. Our results support the strong association of the CUN-BAE index with the incidence of hypertension for the first time in a Mediterranean population.

### 4.2. Mechanisms

Various complex interplaying mechanisms can help explain the hypertension-promoting action of a progressively higher body fat content and obesity, including overactivation of the sympathetic nervous system (SNS) [4,47,48,49,50,51], inappropriate renin–angiotensin–aldosterone system (RAAS) stimulation [5,52,53,54,55,56,57,58,59], modifications induced by adipocyte-derived cytokines (i.e., leptin) [60,61,62,63,64], structural and functional renal changes [65,66,67], and reduced insulin sensitivity [68,69,70,71,72].

Clinical features of SNS hyperactivity include increased heart rate, cardiac output, and renal tubular sodium reabsorption, which have been linked directly to the stimulation of alpha-adrenergic and beta-adrenergic receptors and indirectly by means of the stimulation of other systems, such as the RAAS. Even modest weight gain increases muscle SNS activity [48], which was higher in obese hypertensive patients [49]. Renal SNS activity has been reported to be elevated in obese individuals [50]. In fact, obesity-associated increased SNS activity is variable across different organs, as it predominantly affects kidney and skeletal muscle [4]. However, SNS hyperactivity is not present in all obese patients, and it is influenced by visceral (vs. subcutaneous) adiposity, sex, and ethnicity [51].

Higher levels of all components of the RAAS have been reported in obese vs. lean persons despite obesity-associated volume expansion and sodium retention that normally would downturn the RAAS [52,53]. RAAS activation leads to increased angiotensin II, a potent vasoconstrictor, and stimulates the production of aldosterone. Both molecules increase the renal reabsorption of sodium and the retention of water, provoking the expansion of intravascular volume and consequent hypertension. RAAS activation has a bidirectional interaction with the SNS: the RAAS increases the sympathetic tone, while the SNS activates the RAAS [54]. Activation of the RAAS leads to an increased renin secretion, which is further upregulated due to physical compression of the kidney by the presence of excess visceral and retroperitoneal fat [5]. The macula densa senses the consequent decrease in renal tubular blood flow and sodium delivery and stimulates the secretion of renin through tubule-glomerular feedback [55]. Adipocytes themselves possess an intrinsic RAAS [56]. Remarkably, mice with specific adipocyte deficiency of angiotensin do not develop hypertension while being fed an obesogenic diet [57]. Moreover, adipocytes also produce factors that stimulate the production of aldosterone by the adrenal gland independently of angiotensin II [58,59]. Leptin, a food intake and energy homeostasis regulatory adipokine [60], also simulates SNS activity in the central nervous system exerting pressor effects on the CV system [61]. In normal conditions, leptin overturns appetite and increases energy expenditure. Yet, obese persons have elevated leptin levels without losing weight, indicating a selective leptin resistance [62], which reduces leptin’s appetite-suppressing and metabolic effects without lessening its SNS stimulatory actions [63]. This supports the concept that hyperleptinemia may contribute to obesity-related hypertension [64].

In addition to the mechanical kidney compression, peri-renal fat may induce inflammation and enlargement of the renal medullary extracellular matrix, leading to compression of the renal medulla [65], diminished renal tubular blood flow, and prolonging the time of fractional sodium reabsorption. The consequently decreased sodium delivery to the macula densa stimulates a feedback-mediated reduction in renal afferent arteriolar resistance, which leads to an elevated renal blood flow and secretion of renin from juxtaglomerular cells [5] in an attempt to reestablish the normal sodium delivery to the macula densa. Yet, the increment of glomerular hydrostatic pressure results in progressive glomerular sclerosis and compromises kidney function [66], with an ensuing harmful cycle with injured nephrons, exacerbated sodium retention, and blood pressure rise to maintain the delivery of sodium to the macula densa [67].

Obesity-associated insulin resistance and hyperinsulinemia cause hypertension through several mechanisms. Increased muscle SNS activity [68] and stimulation of paraventricular nucleus (hypothalamic region regulating sympathetic output) [69] have been both observed following systemic insulin infusion. Insulin also promotes renal sodium retention by the activation of the sodium-hydrogen exchanger 3 [70]. Patients with metabolic syndrome, generally considered an insulin-resistant state, have higher fractional sodium reabsorption vs. those without metabolic syndrome [71]. Normally, insulin has vasodilatory effects, but in obese hyperinsulinemic persons, this response is reduced due to endothelial dysfunction, leading to increased vasoconstrictor tone [72].

### 4.3. Strengths and Limitations

Our study has several strengths, including the prospective design, the large size of the sample, the long-term follow-up, the possibility of adjusting for numerous confounders, as well as the high retention rate. Nevertheless, we acknowledge some limitations. First, notwithstanding the SUN cohort characteristics, the application of our results in other populations should be based on shared biological mechanisms and not merely on statistical “representativeness”. Yet, future studies are necessary in order to examine whether our findings are observed in other populations. Second, residual confounding cannot be completely ruled out. Nevertheless, we adjusted our models for a wide array of potential confounders. Third, an additional limitation may be the use of self-reported information. Nonetheless, parameters that were self-reported, i.e., weight, BMI, and hypertension, have been validated in this cohort with good validation results, as shown in previous investigations [32,33,34].

## 5. Conclusions

Increased adiposity assessed with the CUN-BAE index, a validated method to appraise body fat, was strongly, positively, and independently associated with a higher risk of developing hypertension in a relatively young Mediterranean cohort. Even after adjusting for obesity, defined as generally recommended (BMI higher than 30 kg/m^2^), this association remained strongly significant, suggesting that the risk of hypertension starts long before a very high body weight is reached. Our results underscore the crucial role of weight management in reducing and maintaining a low body fat content, even in non-obese persons, to prevent hypertension, a crucial modifiable risk factor for CVD, cerebrovascular events, and kidney failure in all populations.

## Figures and Tables

**Table 1:** Baseline characteristics of men and women according to quartiles (Q) of Body Fat (as captured by the CUN-BAE index) among participants in the SUN (“Seguimiento Universidad de Navarra”) project, 1999–2019 ^1^.

**Men**				
	**Q1**	**Q2**	**Q3**	**Q4**
Limits of body fat (range)	19.5, 32.3	32.3, 35.6	35.6, 38.7	38.7, 58.4
N	1446	1445	1446	1445
Age, y	30.4 (7.3)	37.8 (9.3)	44.0 (10.5)	48.1 (11.7)
Married men, %	27.3	56.0	71.7	78.2
University education, year	5.1 (1.6)	5.5 (1.7)	5.5 (1.8)	5.4 (1.8)
BMI, kg/m^2^	22.1 (1.3)	24.2 (1.0)	25.7 (1.1)	28.8 (2.4)
*Smoking*				
- Never, %	64.2	53.0	40.6	32.0
- Current, %	21.6	21.5	22.1	21.7
- Former smoker, %	14.3	25.5	37.3	46.3
Leisure-time physical activity, METs-h/week	32.8 (32.2)	27.9 (26.1)	25.9 (26.1)	21.1 (20.4)
Television watching, h/day	1.5 (1.2)	1.5 (1.0)	1.5 (1.0)	1.6 (1.1)
Family history of hypertension, %	32.2	37.7	40.2	37.5
Hypercholesterolemia at baseline, %	8.1	15.6	23.7	31.6
Hypertriglyceridemia at baseline, %	2.4	6.0	10.9	19.4
Use of analgesic drugs, %	4.8	7.5	8.8	10.7
Total energy intake, kcal/day	2591 (662)	2481 (633)	2397 (658)	2326 (666)
Adherence to Mediterranean Diet ^2^	4.2 (1.8)	4.5 (1.8)	4.9 (1.8)	5.0 (1.7)
Adoption of special diets, %	3.8	4.6	5.9	7.4
Between-meal snacking, %	32.1	23.8	25.3	27.4
***Dietary consumption***				
Vegetables (g/day)	433 (296)	445 (285)	482 (341)	475 (324)
Fruit (g/day)	265 (229)	287 (249)	331 (315)	314 (312)
Legumes (g/day)	25 (20)	24 (19)	25 (20)	24 (18)
Cereals (g/day)	118 (80)	110 (76)	109 (82)	107 (83)
Whole bread (g/day)	9 (26)	9 (24)	9 (31)	11 (30)
Nuts (g/day)	8 (12)	8 (11)	8 (11)	8 (12)
Olive oil (g/day)	17 (14)	16 (13)	17 (14)	16 (14)
Eggs (g/day)	27 (19)	26 (20)	24 (15)	24 (20)
Fish and other seafood (g/day)	88 (52)	91 (53)	102 (61)	104 (59)
Whole-fat dairy products (g/day)	294 (238)	245 (212)	196 (182)	179 (188)
Low-fat dairy products (g/day)	140 (216)	168 (229)	181 (215)	191 (240)
Meat (g/day)	198 (82)	186 (80)	175 (79)	177 (79)
Coffee (cups/day)	3 (2)	4 (2)	4 (2)	4 (2)
Alcohol	7.6 (8.3)	9.4 (11.9)	10.7 (12.6)	12.1 (14.2)
SSB (servings/day) ^3^	0.3 (0.5)	0.3 (0.5)	0.2 (0.5)	0.2 (0.5)
***Dietary intakes***				
Carbohydrate (% of energy)	44 (7)	43 (7)	44 (8)	43 (8)
Protein (% of energy)	17 (3)	18 (3)	18 (3)	18 (3)
Total fat (% of energy)	37 (6)	36 (6)	35 (6)	35 (6)
MUFAs (% of energy)	15 (3)	15 (3)	15 (3)	15 (4)
SFAs (% of energy)	13 (3)	13 (3)	12 (3)	12 (3)
PUFAs (% of energy)	5 (2)	5 (1)	5 (1)	5 (1)
Vitamin C (mg/day)	245 (144)	245 (129)	259 (146)	248 (139)
Vitamin D (mcg/day)	6.1 (4.0)	6.1 (4.3)	6.3 (4.6)	6.2 (4.3)
Na (mg/day)	3917 (2440)	3619 (2140)	3510 (2444)	3525 (2401)
K (mg/day)	4593 (1443)	4569 (1443)	4650 (1662)	4575 (1636)
Ca (mg/day)	1207 (448)	1199 (468)	1156 (441)	1130 (468)
Mg (mg/day)	415 (119)	410 (118)	412 (129)	405 (127)
Iron from heme sources (mg/day)	17 (5)	17 (5)	17 (5)	17 (5)
Folate (mcg/day)	373 (157)	375 (150)	390 (175)	382 (172)
Dietary fibre (g/day)	26 (11)	26 (11)	27 (13)	26 (12)
**Women**				
	**Q1**	**Q2**	**Q3**	**Q4**
Limits of body fat (range)	27.9, 38.5	38.5, 41.6	41.6, 45.3	45.3, 65.0
N	2542	2543	2541	2542
Age, year	27.0 (5.0)	31.6 (7.1)	36.6 (9.0)	42.2 (10.7)
Married women, %	19.0	37.6	52.4	57.6
University education, year	4.6 (1.2)	4.9 (1.3)	4.9 (1.4)	4.9 (1.4)
BMI, kg/m^2^	19.1 (1.0)	20.8 (0.9)	22.4 (1.1)	25.7 (2.7)
*Smoking*				
- Never, %	60.4	54.8	49.1	44.1
- Current, %	25.7	23.6	22.7	21.1
- Former smoker, %	13.9	21.6	28.2	34.8
Leisure-time physical activity, METs-h/week	19.4 (20.7)	19.7 (20.2)	19.4 (20.7)	17.2 (17.6)
Television watching, h/dat	1.6 (1.4)	1.6 (1.1)	1.6 (1.3)	1.7 (1.2)
Family history of hypertension, %	31.8	39.2	47.2	51.4
Hypercholesterolemia at baseline, %	7.3	9.1	11.7	17.3
Hypertriglyceridemia at baseline, %	1.4	1.6	1.8	4.9
Use of analgesic drugs, %	9.5	11.4	12.6	14.8
Total energy intake, kcal/day	2348 (563)	2320 (575)	2286 (567)	2245 (579)
Adherence to Mediterranean Diet ^2^	4.3 (1.7)	4.4 (1.7)	4.6 (1.7)	4.9 (1.7)
Adoption of special diets, %	4.5	5.5	8.0	14.2
Between-meal snacking, %	38.3	35.1	35.5	40.1
***Dietary consumption***				
Vegetables (g/day)	544 (348)	538 (315)	566 (341)	601 (365)
Fruit (g/day)	338 (291)	345 (286)	374 (290)	400 (327)
Legumes (g/day)	22 (16)	22 (17)	21 (17)	22 (19)
Cereals (g/day)	98 (64)	97 (63)	99 (67)	93 (66)
Whole bread (g/day)	14 (31)	15 (32)	16 (34)	16 (31)
Nuts (g/day)	7 (12)	7 (11)	7 (12)	7 (11)
Olive oil (g/day)	19 (15)	19 (15)	20 (15)	21 (16)
Eggs (g/day)	22 (15)	22 (14)	22 (13)	22 (14)
Fish and other seafood (g/day)	92 (59)	94 (61)	96 (59)	105 (62)
Whole dairy products (g/day)	207 (196)	193 (191)	175 (180)	149 (175)
Low-fat dairy products (g/day)	239 (252)	247 (250)	259 (249)	288 (246)
Meat (g/day)	173 (78)	169 (77)	171 (75)	173 (79)
Coffee (cups/day)	4 (2)	4 (2)	4 (2)	4 (2)
Alcohol (g/day)	3.5 (4.7)	4.1 (5.6)	4.2 (6.4)	4.2 (6.2)
SSB (servings/day) ^3^	0.2 (0.4)	0.2 (0.3)	0.2 (0.3)	0.1 (0.3)
***Dietary intakes***				
Carbohydrate (% of energy)	44 (7)	44 (7)	43 (7)	43 (8)
Protein (% of energy)	18 (3)	18 (3)	18 (3)	19 (4)
Total fat (% of energy)	37 (7)	37 (6)	37 (6)	37 (7)
MUFAs (% of energy)	16 (4)	16 (4)	16 (4)	16 (4)
SFAs (% of energy)	13 (3)	13 (3)	12 (3)	12 (3)
PUFAs (% of energy)	5 (2)	5 (2)	5 (2)	5 (2)
Vitamin C (mg/day)	287 (160)	286 (146)	299 (156)	313 (167)
Vitamin D (mcg/day)	6.0 (4.0)	5.9 (4.2)	6.0 (4.8)	6.4 (4.6)
Na (mg/day)	3313 (2059)	3173 (2020)	3074 (1802)	2985 (2440)
K (mg/day)	4781 (1576)	4747 (1517)	4852 (1551)	4992 (1666)
Ca (mg/day)	1248 (466)	1249 (465)	1256 (472)	1270 (464)
Mg (g/day)	413 (120)	412 (118)	417 (121)	424 (128)
Iron from heme sources (mg/day)	17 (5)	17 (5)	17 (5)	17 (5)
Folate (mcg/day)	415 (173)	413 (168)	423 (177)	440 (191)
Dietary fibre (g/day)	28 (12)	28 (12)	29 (12)	30 (13)

MET: metabolic equivalent task; MUFA: monounsaturated fatty acid; SFA: saturated fatty acid; SSB: sugar sweetened beverages; PUFA: polyunsaturated fatty acid. ^1^ Values are mean (SD) unless otherwise stated. ^2^ Mediterranean Diet Score, 0 to 9 points, according to reference [36]. ^3^ One serving of sugar-sweetened beverages = 200 mL.

**Table 2:** Association between Quartiles of Body Fat (BF), as captured by the CUN-BAE index, and incident hypertension among men and women in the SUN (“Seguimiento Universidad de Navarra”) project, 1999–2019 ^1^.

**Men**	**BF Quartiles**						
	**Q1**	**Q2**	**Q3**	**Q4**	***p*-Trend**	**HR for + 10 Units of BF Increase**	**HR for + 2 Units of BF Increase**
*n*	1446	1445	1446	1445			
Median (p25, p75)	30.1 (28.4, 31.3)	34.1 (33.2, 34.8)	37.1 (36.4, 37.8)	40.8 (39.6, 42.5)			
Incident hypertension (cases)	123	223	391	529			
Person-years of follow-up	17,061	17,802	17,501	16,480			
Crude rate (×10^−3^)	7.2	12.5	22.3	32.1			
Crude HR	1 (ref.)	1.69 (1.36, 2.11)	3.30 (2.70, 4.04)	5.49 (4.51, 6.69)	<0.001	3.47 (2.87, 4.21)	1.28 (1.23, 1.33)
Multivariable-adjusted HR ^2^ Model 1	1 (ref.)	1.42 (1.13, 1.77)	2.31 (1.86, 2.87)	3.30 (2.64, 4.11)	<0.001	3.16 (2.62, 3.81)	1.26 (1.21, 1.31)
Multivariable-adjusted HR ^3^ Model 2	1 (ref.)	1.41 (1.13, 1.77)	2.31 (1.86, 2.87)	3.19 (2.55, 4.00)	<0.001	3.35 (2.65, 4.24)	1.27 (1.22, 1.33)
**Repeated measures**							
Crude HR	1 (ref.)	1.79 (1.43, 2.25)	3.07 (2.49, 3.79)	5.24 (4.29, 6.40)	<0.001	3.36 (2.81, 4.02)	1.27 (1.23, 1.32)
Multivariable-adjusted HR ^2^ Model 1	1 (ref.)	1.51 (1.19, 1.90)	2.32 (1.86, 2.89)	3.37 (2.71, 4.19)	<0.001	3.24 (2.67, 3.94)	1.26 (1.22, 1.32)
Multivariable-adjusted HR ^3^ Model 2	1 (ref.)	1.50 (1.19, 1.89)	2.31 (1.85, 2.88)	3.15 (2.51, 3.95)	<0.001	3.72 (2.86, 4.84)	1.30 (1.23, 1.37)
**Women**							
*n*	2542	2543	2541	2542			
Median	36.6 (35.2, 37.6)	40.1 (39.3, 40.9)	43.4 (42.5, 44.3)	48.0 (46.5, 50.1)			
Incident hypertension (*n*)	78	136	222	258			
Person-years	28,936	29,212	29,440	28,337			
Crude rate (×10^−3^)	2.7	4.7	7.5	16.1			
Crude HR	1 (ref.)	1.70 (1.29, 2.25)	2.90 (2.24, 3.75)	7.07 (5.56, 8.99)	<0.001	3.58 (3.04, 4.20)	1.29 (1.25, 1.33)
Multivariable-adjusted HR ^2^ Model 1	1 (ref.)	1.52 (1.15, 2.02)	2.18 (1.67, 2.85)	4.50 (3.46, 5.85)	<0.001	3.28 (2.75, 3.92)	1.27 (1.22, 1.31)
Multivariable-adjusted HR ^3^ Model 2	1 (ref.)	1.52 (1.15, 2.02)	2.18 (1.66, 2.85)	4.35 (3.34, 5.68)	<0.001	3.53 (2.86, 3.36)	1.29 (1.23, 1.34)
**Repeated measures**							
Crude HR	1 (ref.)	1.81 (1.35, 2.42)	3.19 (2.44, 4.16)	6.83 (5.33, 8.75)	<0.001	3.08 (2.61, 3.64)	1.25 (1.21, 1.29)
Multivariable-adjusted HR ^2^ Model 1	1 (ref.)	1.61 (1.21, 2.16)	2.59 (1.97, 3.40)	4.72 (3.63, 6.14)	<0.001	3.16 (2.68, 3.73)	1.26 (1.22, 1.30)
Multivariable-adjusted HR ^3^ Model 2	1 (ref.)	1.61 (1.20, 2.16)	2.58 (1.96, 3.39)	4.26 (3.26, 5.57)	<0.001	3.20 (2.63, 3.89)	1.26 (1.21, 1.31)

^1^ Values were HR estimated with Cox regression and 95% confidence intervals (CI). ^2^ Model 1: HR adjusted for marital status, body weight changes, years of university education, smoking, pack-year, physical activity, television watching, family history of hypertension, total energy intake, adherence to the Mediterranean diet, sugar-sweetened beverages, between-meal snacking, adoption of special diet, dietary sodium, dietary potassium, dietary calcium, dietary magnesium, hypercholesterolemia, hypertriglyceridemia, use of analgesic drugs, year of entrance to the cohort. ^3^ Model 2: HR adjusted for factors in Model 2 plus BMI higher than 30 kg/m^2^.

**Table 3:** Association between z-BF (i.e., for each SD, as a continuous variable) and incident hypertension among men and women in the SUN (“Seguimiento Universidad de Navarra”) project, 1999–2019 ^1^.

z-BF	Men	*p*	Women	*p*
*n*	5783		10,168	
Persons year	68,844		115,954	
Crude rate (×10^−3^)	18.4		7.7	
Crude HR	1.90 (1.79, 2.01)	<0.001	2.02 (1.91, 2.15)	<0.001
Multivariable-adjusted HR ^2^ Model 1	1.58 (1.47, 1.69)	<0.001	1.72 (1.60, 1.85)	<0.001
Multivariable-adjusted HR ^3^ Model 2	1.72 (1.57, 1.87)	<0.001	1.80 (1.66, 1.97)	<0.001
**Repeated measures**				
Crude HR	1.75 (1.66, 1.85)	<0.001	1.92 (1.82, 2.04)	<0.001
Multivariable-adjusted HR ^2^ Model 1	1.51 (1.42, 1.60)	<0.001	1.72 (1.61, 1.84)	<0.001
Multivariable-adjusted HR ^3^ Model 2	1.62 (1.49, 1.76)	<0.001	1.78 (1.63, 1.94)	<0.001

^1^ Values were HR estimated with Cox regression and 95% confidence intervals (CI). ^2^ Model 1: HR adjusted for marital status, body weight changes, years of university education, smoking, pack-year, physical activity, television watching, family history of hypertension, total energy intake, adherence to the Mediterranean diet, sugar-sweetened beverages, between-meal snacking, adoption of special diet, dietary sodium, dietary potassium, dietary calcium, dietary magnesium, hypercholesterolemia, hypertriglyceridemia, use of analgesic drugs, year of entrance to the cohort. ^3^ Model 2: HR adjusted for factors in Model 2 plus BMI higher than 30 kg/m^2^.

**Table 4:** Sensitivity analyses: Multivariable-adjusted Hazard Ratios of incident hypertension associated with Body Fat (BF), as captured by the CUN-BAE index, among men and women in the SUN (“Seguimiento Universidad de Navarra”) cohort, 1999–2019 ^1^.

**Men**	***n***	**Cases of Incident Hypertension** ***n***	**HR (95% CI) ^2,3^**
Main analysis ^2,3^	5782	1266	3.19 (2.55, 4.00)
Changing allowable energy limits (percentiles 1–99) ^2,3,4^	6105	1342	2.95 (2.38, 3.67)
Censoring follow-up at ≥ 14 year ^2,3^	5782	1144	3.69 (2.56, 5.34)
Excluding early incident hypertension (first 2 year) ^2,3^	5491	975	3.01 (2.34, 3.87)
Including only participants < 40 year ^2,3^	3076	397	3.63 (2.52, 5.24)
Including only participants < 60 year ^2,3^	5487	1144	3.14 (2.49, 3.96)
BF in quintiles (Q5 vs. Q1) ^2^	5782	1266	3.67 (2.82, 4.78)
**Women**			
Main analysis ^2,3^	10,168	894	4.35 (3.34, 5.68)
Changing allowable energy limits (percentiles 1–99) ^2,3,4^	11,197	978	4.14 (3.23, 5.32)
Censoring follow-up at ≥ 14 year ^2,3^	10,168	785	5.25 (3.47, 7.96)
Excluding early incident hypertension (first 2 year) ^2,3^	9981	707	4.45 (3.30, 6.01)
Including only participants < 40 year ^2,3^	7391	419	2.95 (2.13, 4.09)
Including only participants < 60 year ^2,3^	10,067	856	4.08 (3.12, 5.33)
BF in quintiles (Q5 vs. Q1) ^2^	10,168	894	4.31 (3.23, 5.75)

^1^ Values were HR estimated with Cox regression and 95% confidence intervals (CI). ^2^ HR adjusted for marital status, body weight changes, years of university education, smoking, pack-year, physical activity, television watching, family history of hypertension, total energy intake, adherence to the Mediterranean diet, sugar-sweetened beverages, between-meal snacking, adoption of special diet, dietary sodium, dietary potassium, dietary calcium, dietary magnesium, hypercholesterolemia, hypertriglyceridemia, use of analgesic drugs, year of entrance to the cohort, BMI higher than 30 kg/m^2^. ^3^ Q4 vs. Q1. ^4^ The sample size of this sensitivity analysis (changing allowable energy limits) was larger than the sample size of the main analysis because changing these allowable limits inherently led to a different sample size.

## Data Availability

The data that support the findings of this study are available from the SUN Project at sun@unav.es, upon reasonable request.
